# Cubitus varus: l'ostéotomie de soustraction externe a-t-elle toujours sa place? Etude rétrospective à propos de 25 cas

**DOI:** 10.11604/pamj.2016.23.263.5296

**Published:** 2016-04-29

**Authors:** Aniss Chagou, Réda Allah Bassir, Abdelkarim Rhanim, Abdou Lahlou, Mohammed Saleh Berrada, Moradh Yaacoubi

**Affiliations:** 1 Service de Traumatologie-Orthopédie, Centre Hospitalier Universitaire de Rabat, Université Mohammed V, Rabat, Maroc

**Keywords:** Cubitus varus, cal vicieux, soustraction externe, Cubitus varus, malunion, external subtraction

## Abstract

Le cubitus varus est cal vicieuxle plus souvent secondaire à des fractures supracondyliennes déplacées de l'extrémité inférieure de l'humérus chez l'enfant. Différentes techniques chirurgicales ont été proposées avec un taux de réussite différents mais aussi un taux de complications rapportées différents. Nous évaluons à travers une étude rétrospective de 25 cas colligés au centre hospitalier universitaire de Rabat, les résultats de la technique de soustraction externe que nous utilisons dans notre formation pour prendre en charge cette déformation.

## Abstract (en)

Cubitus varus or malunion is most often secondary to displaced supracondylar fractures of the lower end of the humerus in children. Various surgical techniques have been proposed with different success rates but also with different reported complication rates. Our retrospective study of 25 cases diagnosed at the University Hospital of Rabat evaluated the results of the technique of external subtraction which was used in our training to handle this deformation.

## Introduction

Le cubitus varusreprésente un cal vicieux en varus, consécutif aux fractures supra condyliennes, généralement déplacées de l'extrémité inférieure de l'humérus. Il a pour cause soit une mal unionsupra condylienne soit une ostéonécrose de la trochlée. Les composantes de la déformation sont un varus, une rotation interne et une hyper extension.

Le cubitus varus n'altère que peu la fonction du membre, l'indication est posée pour la gène esthétiqueoccasionnéemais aussi pour éviter certaines complications telle que la paralysie nerveuse ulnaire, l'instabilité rotatoire postéro latérale et la fracture secondaire de l'humérus distal.

Un grand nombre de méthodes de correction ont été décritesdont la soustraction externe, la fermeture interne, l'ostéotomie en dôme, l'ostéotomie tridimensionnelle et l'ostéotomie step-cut. Par ailleurs, un grand nombre de moyens de fixation ont été utilisés: le vissage, laplaque vissée, l'embrochage. Parallèlement à cet arsenal thérapeutique, un grand nombre de complications ont été reportées.

A travers une étude rétrospective portant sur 25 cas colligés au centre hospitalier universitaire de Rabat entre 2004 et 2013présentant un cubitus varus séquellaire de fractures supracondyliennes du coude ayant tous bénéficié d'une ostéotomie supracondylienne de soustraction externe. Nous évaluons le résultat de cette technique de soustraction mais aussi du moyen de fixation, après un recul de allant de 6 à 10 ans.

## Patient et observation

Il s'agit d'une étude rétrospective incluant 25 adultes jeunes opérés au service de traumatologie-orthopédie du centre hospitalier universitaire de Avicenne de Rabat entre 2004 et 2013. Les cubitus varus opérés sont tous séquellaires de fractures supra condyliennes de l'extrémité inférieure de l'humérus de stade 3 dans l'enfance. Les patients ont été suivis cliniquement et radiologiquement avec un recul de 38 mois en moyenne.

L’âge de survenue des fractures est de 2 à 9 ans avec une moyenne de 5,2 ans. Tous les patients avaient bénéficié d'un traitement orthopédique et la déformation est apparue dans les 30 mois après le traitement en moyenne. Nous avons posé l'indication devant un varus de 15° minimum, la mobilité n'a jamais été le motif de l'opération mais plutôt le préjudice esthétique.

Des radiographies préopératoires ont été réalisées afin de définir l'angle à soustraire, et la zone de l'ostéotomie. Cette dernière a toujours été abordée par une voie latérale sur le tiers inférieur de l'humérus. Nous avons réalisé à chaque fois une ostéotomie de soustraction à la jonction diaphyso-métaphysaire selon la technique de Descamps tout en essayant de restaurer le valgus physiologique.

**Figure 1:**
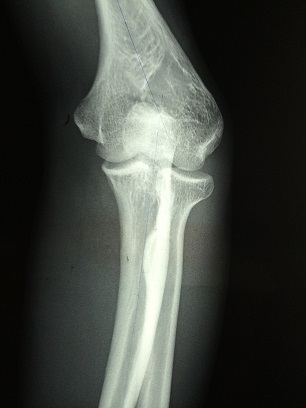
Radiographie de face montrant la deformation en varus

**Figure 2:**
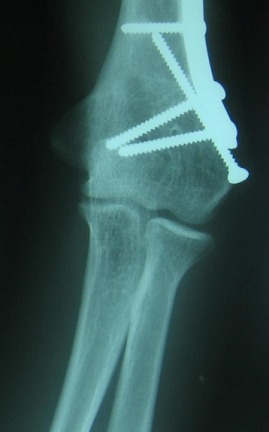
Radiographie de face montrant la correction de la fixation par vissage et plaque vissée

**Figure 3:**
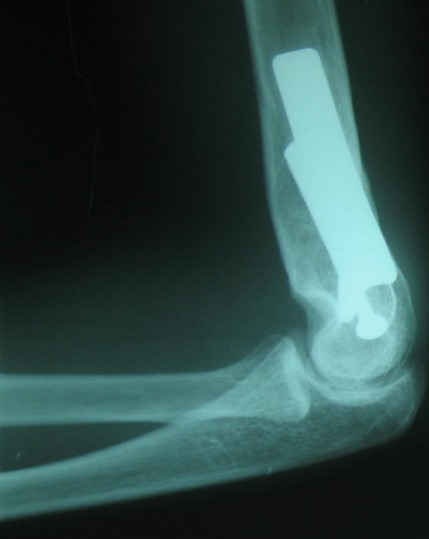
Radiographie de profil du même patient

**Figure 4:**
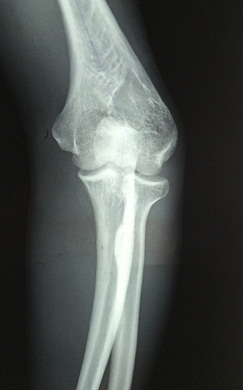
Radiographie de face montrant la déformation en varus du deuxième patient

**Figure 5:**
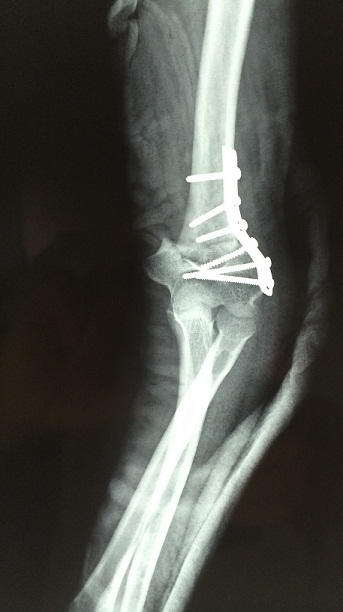
Radiographie de face montrant une fixation par plaque vissée, l'ostéosynthèse est restée stable même si l'ostéotomie a entamé la corticale interne

La fixation a été assurée dans 9 cas par une seule vis, dans 7 cas par deux vis, dansun cas par une plaque vissée de neutralisation et une vis (Figure 1, Figure 2 et Figure 3) et dans 8 cas par une plaque vissée (Figure 4 et Figure 5). Nous avons effectué une translation interne associée chez 8 patients.

Les résultats ont été jugés radiologiquement après un recul de 6 à 10 ans et ont été classés de la façon suivante, très bon si restauration du valgus physiologique, bon si restauration d'un valgus ou sujet hyper ou hypo corrigé (entre 5°et 15°). mauvais si varus supérieur à 5°.

En suivant ces critères nos résultats ont été jugés très bons résultats chez 5 patients, bons résultats chez 16 patients et mauvais résultats chez 4 patients. Nous avons eu deux infections superficielles ayant bien évolué sous antibiothérapie et soins locaux. Nous avons pas relevé de complications nerveuses. La consolidation s'est faite dans les temps sauf pour 4 patients où nous avons été contraints de prolonger l'immobilisation pendant 2 semaines supplémentaires. Nous avons malheureusement déploré 4 cas d’échecs du traitement que nous incriminons au défaut de résection du coin pour 3 cas et un cas d’échec du à une ostéosynthèse instable secondaire à une ostéotomie entamant la corticale interne.

## Discussion

L'objectif essentiel de la chirurgie du cubitus varus post-traumatique est l'obtention d'une apparence normale et d'un aspect esthétique satisfaisant du membre [1, 2]. Toutefois les ostéotomies de correction sont de leur part associées à de multiples complications esthétiques [3]: proéminence latérale de l'humérus distal faisant suite à une ostéotomie de soustraction externe, une cicatrice hypertrophique faisant suite à un abord latéral [2], une récidive de la déformation en varus [4] surtout quand la déformationrotationnelle est corrigée ou quand la fixation n'est pas solide.

D'autres complications ont été décrites, des paralysies nerveuses iatrogènes radiale et cubitale [4, 5], des instabilités postéro latérales et latérales iatrogènes [6] et aussi des infections postopératoires. Yun [7] a rapporté une série de 22 cas en 2007 utilisant une ostéotomie en coin avec d'excellents résultats et 2 complications, une fracture peropératoire et une paralysie ulnaire. kim et al ont utilisé la même technique et ont rapporté des résultats satisfaisants avec un seul cas de paralysie ulnaire. De Roza et Graziano [8] ont traité 11 patients par la technique de stepcut et n'ont fait face à aucune complication mais un de leurs patients avait toujours un varus résiduel. Matsushita and Nagano [5] ont suivi 12 patients après une ostéotomie en arc et n'ont observé aucune complication. Ipolito et al [9] sur une série de 24 cas ont rapporté 6 complications immédiates dont une paralysie nerveuse ulnaire, il a noté également après un recul de 23 ans que quatorze patients étaient insatisfaits du résultat et 12 avait une atrophie notable. Oppeinheim et al [10] ont rapporté un taux de complications de 24% incluant des neuropraxies et aspect esthétique insatisfaisant.

Notre série a objectivé 21 cas de résultats satisfaisants pour 25 patients opérés, nous n'avons pas fait face à des complications nerveuses. Nous pouvons dire suivant les résultats de notre série que l'ostéotomie selon la technique de Descamps a sa place dans l'arsenal thérapeutique du cubitus varus chez l'adulte jeune. Elle permet d'atteindre une correction satisfaisante avec un taux de complications faible. Il faut aussi souligner la nécessité de mettre en place une fixation solide, une fixation par 2 vis ou une plaque paraîtraisonnable. Il n'en reste pas moins que l'ostéotomie de soustraction externe est une technique délicate notamment par la difficulté d'appréciation du coin à réséquer. L'indication opératoire doit rester prudente. Il s'agit d'une opération à visée essentiellement esthétique, elle doit être posée lorsqu'elle est souhaitée par le patient et pour un varus de 15° au moins.

## Conclusion

Le cubitus varus séquellaire des fractures supra condyliennes est un cal vicieux résultant de mal union supracondylienne ou ne nécrose de la trochlée. L'ostéotomie de soustraction à une place importante dans l'arsenal thérapeutique, elle permet de rétablir l'axe du membre avec un taux faible de complications nerveuses. Toutefois l'indication doit rester prudente et bien pesée; le but étant essentiellement esthétique la décision doit être prise après concertation avec le patient et pour des varus supérieur à 15°.
